# Gel Polymer Electrolytes Based on Silica-Added Poly(ethylene oxide) Electrospun Membranes for Lithium Batteries

**DOI:** 10.3390/membranes8040126

**Published:** 2018-12-05

**Authors:** Maria Assunta Navarra, Lucia Lombardo, Pantaleone Bruni, Leonardo Morelli, Akiko Tsurumaki, Stefania Panero, Fausto Croce

**Affiliations:** 1 Dipartimento di Chimica, Sapienza Università di Roma, Piazzale Aldo Moro 5, 00185 Rome, Italy; lucia.lombardo@uniroma1.it (L.L.); morelli.1424793@studenti.uniroma1.it (L.M.); akiko.tsurumaki@uniroma1.it (A.T.); stefania.panero@uniroma1.it (S.P.); 2 Dipartimento di Farmacia, Università “G. d’Annunzio” Chieti-Pescara, Via dei Vestini 31, 66100 Chieti, Italy; pantaleone.bruni@unich.it

**Keywords:** gel polymer electrolyte, electrospinning, lithium batteries

## Abstract

Solid polymer electrolytes, in the form of membranes, offering high chemical and mechanical stability, while maintaining good ionic conductivity, are envisaged as a possible solution to improve performances and safety in different lithium cell configurations. In this work, we designed and prepared systems formed using innovative nanocomposite polymer membranes, based on high molecular weight poly(ethylene oxide) (PEO) and silica nanopowders, produced by the electrospinning technique. These membranes were subsequently gelled with solutions based on aprotic ionic liquid, carbonate solvents, and lithium salt. The addition of polysulfide species to the electrolyte solution was also considered, in view of potential applications in lithium-sulfur cells. The morphology of the electrospun pristine membranes was evaluated using scanning electron microscopy. Stability and thermal properties of pristine and gelled systems were investigated uisng differential scanning calorimetry and thermal gravimetric analysis. Electrochemical impedance spectroscopy was used to determine the conductivity of both swelling solutions and gelled membranes, allowing insight into the ion transport mechanism within the proposed composite electrolytes.

## 1. Introduction

Intense research efforts are still directed to improve the characteristics of lithium battery devices for energy storage. In particular, attention is directed to those devices that possess the requirements of safety and performance for powering electric vehicles [1] or for stationary use in smart-grids [2,3]. In this scenario, great advances in terms of reliability can be achieved by moving from liquid to polymer electrolytes. Among the different types of polymer electrolytes for lithium batteries, those based on polyethylene oxide (PEO) membranes, have received significant attention [4].

PEO membranes combine high chemical stability, solid-like diffusivity, and good ionic conductivity [5,6]. The main problem that has limited the use of PEO is its poor ionic conductivity at room temperature (10^−8^ S/cm) due to the presence of crystalline phases. Conductivity values of interest for applications in lithium batteries (>10^−4^ S/cm) are reached only above 65 °C, i.e., beyond the melting temperature of the polymer crystalline phase. The reason for this behavior is to be found in the peculiar PEO conduction mechanism as the lithium ion moves from a coordination center (the PEO oxygen atoms) to the next. This movement is made possible only if the chain undergoes subsequent rearrangement steps. The dynamics of this process is greatly facilitated by a high mobility of the chains. Consequently, high values of ionic conductivity are reached above the melting temperature in the amorphous phase where long-range chain active motions are possible [7].

In this paper we address the problem of PEO low room temperature conductivity using three different strategies [8]. The first one consists of incorporating inside the fibers of electrospun PEO membranes inert ceramic particles, which act as fillers. The presence of these particles inhibits the PEO chains tendency to crystallize, leading to membranes with a higher amorphous fraction. In addition, as is well documented in the literature, it is known that the fillers stabilize the lithium electrolyte interface and increase the Li^+^ transference number due to Lewis acid–base type interactions between the ceramic surface groups and the polymer chains coordination sites. As an additional benefit, the presence of this inorganic dispersion improves the mechanical properties of the composite polymer membranes. In general, the best overall performances have been obtained with nanometric particles having acidic surface groups and in a 5–20 wt.% ratio with respect to the polymer [5,8,9,10,11]. The second strategy involves the use of lithium salts with low lattice energy, which favors the lithium salt dissociation. To achieve this goal, lithium salts with large and flexible anions, that disperse effectively the charge and are able to increase the free-volume between the polymer chains and thus to promote the ionic mobility, have been used [8,12,13]. The third approach has involved the addition of an aprotic ionic liquid (IL) that increases the total ionic conductivity, competing with lithium-ions toward the binding sites of coordination and creating additional free-volume between the chains [8]. The disadvantages of ionic liquids are their high viscosity, their tendency to form ionic clusters, and their high cost [14]. To mitigate such drawbacks, we have added to the electrolyte formulations alkyl carbonates, which reduce the viscosity of the solution and form a protective passivating film (i.e., a solid–electrolyte interface, SEI) on the lithium anode [8].

In this perspective, we designed and prepared innovative electrolytic systems with the aim of providing a compositional study and elucidation on the role of different components. Emphasis is given to the analysis of the conducting properties of the proposed systems as a figure of merit in view of applications in electrochemical devices. Such electrolytes consist of nanocomposite polymer membranes of PEO/silica produced through the electrospinning technique and then gelled using two liquid solutions. The two solutions, based on the aprotic ionic liquid PYR_14_TFSI, contain a mixture of two carbonate solvents, i.e., ethylene carbonate and dimethyl carbonate (EC and DMC), and the bis(trifluoromethane)sulfonyl imide lithium salt (LiTFSI). In one of the two solutions, the polysulfide Li_2_S_8_ was also added. The addition of this last component is justified by the fact that these systems have been thought to provide possible future applications not only in conventional lithium batteries but, specifically, in lithium-sulfur batteries [15,16,17]. The dissolution of the sulfur cathode in the form of polysulfides (and the consequent shuttle phenomenon) strongly limits the development of this technology. For this reason, we intend to reduce such dissolution playing on the solubility equilibrium, by adding a buffer polysulfide to the electrolyte [18,19,20]. Also, some ILs (such as *N*-methyl-*N*-butylpyrrolidinium bis(trifluoromethanesulfonyl)imide, PYR_14_TFSI) have already demonstrated the ability to suppress the dissolution of polysulfides increasing the performance of sulfur cathodes [16]. The choice of LiTFSI as lithium source is due to its stability, large dimension, and flexible imide structure. These latter properties, together with the strong electron withdrawing behavior of the (trifluoromethane)sulfonyl group, enhance the negative charge delocalization and, in turn, guarantee a high salt dissociation level. Moreover, the TFSI anion is the same contained in the ionic liquid, which should avoid the Li^+^-ion transference number reduction, possibly occurring when additional ionic species are added to the solution [21].

## 2. Experimental

### 2.1. Materials for Membranes Preparation

Poly(ethylene oxide) (PEO, Mw = 4 M ≈ 4 × 10^6^ g/mol) and Silica (Nanopowder, average size = 10–20 nm—“BET” Brunauer–Emmett–Teller) were purchased form Sigma-Aldrich (St. Louis, MO, USA). Bi-distilled water was produced in house.

### 2.2. Membrane Separators Fabrication

An in-house made electrospinning apparatus, composed of a high voltage power supply (Spellman SL 50 P 10/CE/230, Hauppauge, NY, USA), a syringe pump (KD Scientific 200 series, Holliston, MA, USA), a glass syringe, a stainless steel blunt-ended needle (inner diameter: 0.84 mm) connected with the power supply electrode, and a grounded aluminum plate-type collector (area ≈10 cm^2^), was utilized in order to prepare the electrospun PEO membranes. The water-based PEO polymer solution was dispensed through a Teflon tube to the needle that was vertically placed on the collecting plate. PEO was dissolved at a concentration of 3% w/v in bi-distilled water. To create the composite systems, after the polymer dissolution, silica nanoparticles were added to the resulting solutions in proper amounts in order to produce a final membrane containing 10% w/w of silica. The realized dispersions were electrospun by using the following conditions: voltage = 13 kV, needle-to-collector distance = 30 cm, and flow rate = 0.005 mL/min. A quality control was performed in order to verify the homogeneity of the sample thickness by means of a digital micrometer. Only specimens having thicknesses in the range of 50 ± 10 μm were used for further characterizations.

The two synthesized membranes will be here labelled as “es-PEO” (for the electrospun sample based on pure PEO) and “es-PEO-SiO_2_” (for the electrospun sample based on PEO with 10% of silica particles)

### 2.3. Swelling Solutions

Liquid electrolyte solutions were prepared as gelling media of the polymer membranes, in order to evaluate the potentialities of the resulting gelled membranes as polymer electrolytes for lithium batteries. In particular, in view of their potential application in lithium-sulfur batteries, two similar solutions, with and without a polysulfide (henceforward called “PS-containing sol” and “PS-free sol,” respectively), were prepared. PS-containing sol was formed using PyR_14_TFSI 77 wt.%, EC:DMC (1:1 volume ratio) 18.4 wt.%, LiTFSI 0.5 mol/kg, Li_2_S_8_ 4.6 wt.%, and PS-free sol used PyR_14_TFSI 80 wt.%, EC:DMC (1:1 volume ratio) 20 wt.%, and LiTFSI 0.5 mol/kg.

The ionic liquid N-methyl-N-butylpyrrolidinium bis(trifluoromethanesulfonyl)imide (PYR_14_TFSI) was bought from Solvionic (99.9%, Toulouse, France) and used as received; the lithium salt (bis(trifluoromethane)sulfonyl imide (LiTFSI) was purchased from Fluka (99.9%, Munich, Germany) and used without further purification; carbonates solvents, i.e., ethylene carbonate (EC) and dimethyl carbonate (DMC), were provided by Sigma-Aldrich. Polysulfide (in form of Li_2_S_8_) was produced in situ via direct reactions between metallic lithium (Chemetall, Frankfurt am Main, Germany) and elemental sulfur (Sigma-Aldrich) in stoichiometric ratio, in a corked vial where the other components were already presents; the mixture was heated to 60–70 °C, stirred for the first four hours, and let it stand for the next twenty hours. These operations were performed in a controlled argon atmosphere dry box having a humidity content below 1 ppm.

### 2.4. Characterizations

The PEO-based membranes were characterized, both dry and after gelation by the two liquid electrolytes.

Gelation of membranes was realized by dropping the liquid electrolyte on disks of membranes, keeping the weight ratio solution-to-membrane ≈5.

The morphology of the electrospun pristine dry membranes was evaluated by means of scanning electron microscopy (SEM—EVO50, Zeiss, Jena, Germany). In order to reduce the charge accumulation, the membranes were covered with a thin layer of evaporated gold before SEM measurements.

Thermal properties were evaluated by means of differential scanning calorimetry (DSC) and thermal gravimetric analysis (TGA). DSC measurements on both pristine and gelled membranes, as well as on pure PEO powder, were performed with a Mettler-Toledo DSC 821 (Zaventem, Belgium) instrument under an inert nitrogen flux, cooling from room temperature down to −90 °C, holding for 10 min at −90 °C, and then heating up to 80 °C at a scan rate of 10 °C/min. TGA was carried out on pristine membrane samples and on pure PEO powder, with a Mettler-Toledo TGA/SDTA 851e under an inert nitrogen flux, heating from room temperature to 600 °C at a scan rate of 10 °C/min.

Electrochemical impedance spectroscopy (EIS) was used to determine the conductivity of the two swelling solutions and of the gelled membranes. To obtain the conductivity values of the solutions, the measurements were carried out by dipping a conductivity cell for liquids (composed of two sheets of platinum facing at the distance of 1 cm) in the test solution and controlling the temperature with a oven (Büchi-Oven B-585, BUCHI Italia S.r.l., Cornaredo, Italy) in the range 20–60 °C. A VSP potentiostat/galvanostat (Bio-Logic Science Instruments, Seyssinet-Pariset, France) was used to record the impedance spectra of the samples in the frequency range of 1 mHz–1 kHz with a sinusoidal signal of 5 mV amplitude. All the spectra, plotted as Nyquist plots, showed an almost vertical straight line intercepting the real axis at high frequency. The value in Ω of this intercept, i.e., the cell resistance at infinite frequency, has been used to calculate the conductivity of the samples under study through the equation:

σ = 1/R × (L/S),

where “L” and “S” are, respectively, the distance in centimeters and the surface area in centimeters square of the electrodes.

The conductivity of gelled membranes was evaluated using EIS, assembling coin-type cells with stain-less steel current collector electrodes, where the swollen PEO membrane acts as an electrolyte separator. For each cell, a 100 µm-thick Teflon O-ring spacer was adopted and two disks of membrane having diameter 0.8 cm were directly gelled in the cell by dropping the desired amount of liquid electrolyte. Impedance spectra were recorded by applying a 10 mV amplitude signal in the frequency range 200 kHz–1 kHz using a VMP2 potentiostat/galvanostat (Bio-Logic Science Instruments, Seyssinet-Pariset, France). The temperature was controlled in the range −50 °C to +60 °C with a Tenney Junior Compact Temperature Test Chamber (TPS, White-Deer, PA, USA).

## 3. Results and Discussion

**Figure 1:**
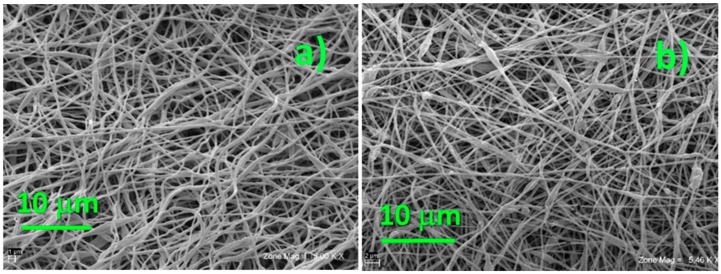
SEM images of the two electrospun membranes: (**a**) without silica additive, and (**b**) with 10 wt.% SiO_2_.

Morphology of pristine PEO-based electrospun membranes is shown in Figure 1. SEM images were recorded for both es-PEO (Figure 1a) and es-PEO-SiO_2_ (Figure 1b) samples, without and with the inorganic additive, respectively. All samples are made of bead-free fibers with an average diameter of 250–300 nm. When nanoparticles were added to PEO solution, the resulting fibers showed slightly higher diameter that can be explained by the inclusion of silica particles inside the polymer fibers. Moreover, Figure 1b shows a certain degree of silica agglomerates, deposited on single fibers or within voids in the polymer mat. Such micrometric agglomerates resulted from the aggregation during the electrospinning process in aqueous media of silica particles, given as spherical porous nanopowder by the product specification.

**Figure 2:**
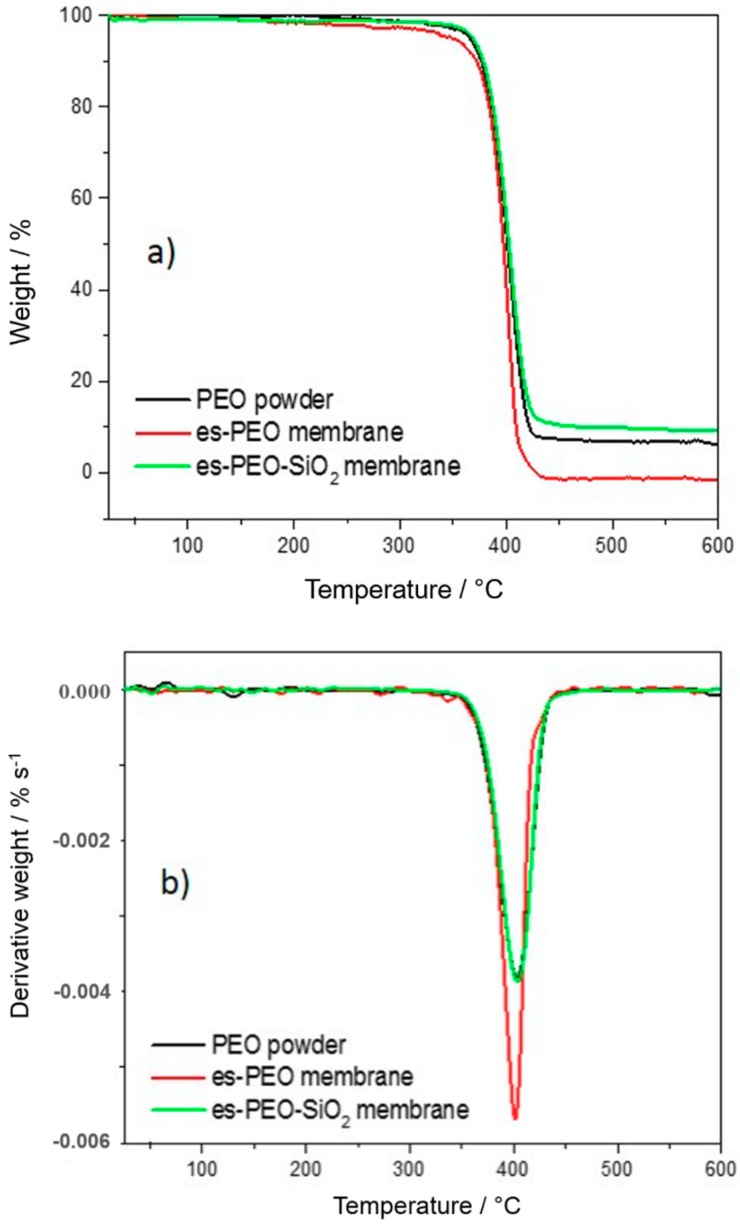
TGA (**a**) and derivative-TGA (**b**) curves of the two electrospun pristine membranes and of pure PEO powder.

Thermal stability of pristine membranes, compared to pure PEO powder, was investigated by TGA measurements, as shown in Figure 2.

High thermal stability typical of pure PEO polymer, extending up to 400 °C, is preserved in both pristine membranes. As highlighted by the minimum of the derivative curves in Figure 2b, temperatures of decomposition were quite similar for all the investigated samples (i.e., 401 °C for the es-PEO membranes and ≈404 °C for both PEO powder and es-PEO-SiO_2_). Unexpected residual masses were revealed above 450 °C in Figure 2a. Pure PEO powder was not completed removed beyond its decomposition temperature, giving ≈8% of left over weight. This could be attributed to non-volatile residuals and to the presence of thermo-stable additives or catalytic compounds used in the synthesis of the polymer. It should be noticed that such residual mass is expected to be reduced by lowering the heating rate, due to a diffusion-limited elimination of the decomposition products by the powder bulk. This did not happen in the case of es-PEO membrane, showing no residuals after it decomposes. Products of decomposition were easily removed from this high surface area sample and eventual additives, such as inhibitors and stabilizers, present in the starting powder were separated and eliminated during the electro-spinning process in aqueous media. A residual mass slightly higher than 10% was revealed for the es-PEO-SiO_2_ membrane, which was attributed to the inorganic silica filler.

**Figure 3:**
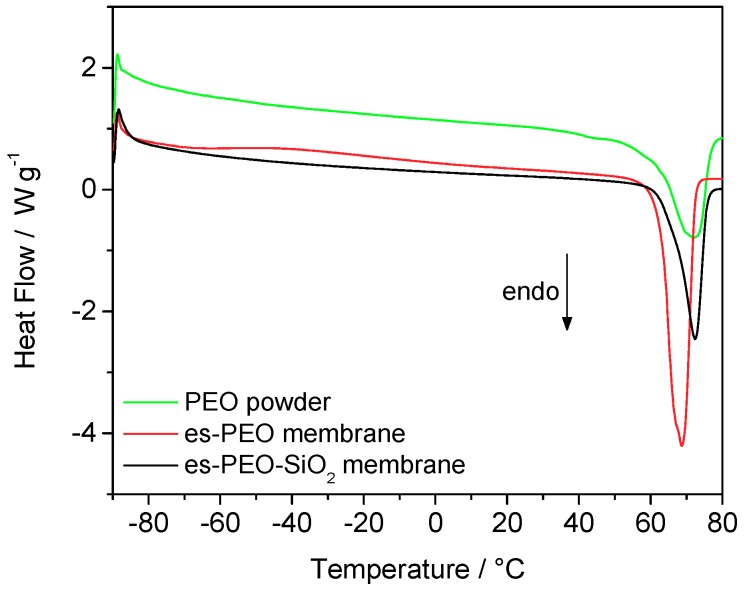
Heating scan of the DSC curves recorded for the two electrospun pristine membranes and for pure PEO powder.

The DSC response of the two pristine membranes, compared with pure PEO powder, is displayed in Figure 3. One main thermal transition was evident in all the DSC traces due to the melting of the polymer crystalline phase. The temperature and energy involved in this melting process have been evaluated and reported in Table 1. Temperature values here shown have been derived from the minimum of the endothermic peak, and in this respect, no valuable differences are noticed among the samples. The additive-free electrospun membrane is actually the sample showing a slightly lower melting temperature and a narrower peak. More remarkable differences were observed in terms of the enthalpy change, highlighting the role of both the electrospinning process and the silica filler. Clearly, lower energy was involved in the melting of pure PEO powder, which also revealed its lower crystallinity. The electrospinning process, due to its ordering effect, somehow increased the crystalline degree of the membranes, compared to the starting polymer powder, corresponding to a higher melting enthalpy. On the contrary, silica nanoparticles and their micrometric aggregates have the effect of lowering crystallinity, towards a more amorphous system with respect to plain, PEO-based electrospun membranes. 

**Figure 4:**
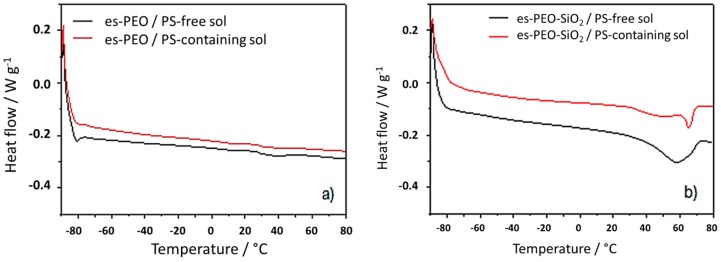
Heating scan of the DSC response of additive free PEO membrane (**a**) and of silica-added PEO membrane (**b**) gelled using liquid electrolyte “PS-free sol” (black curves) or “PS-containing sol” (red curves).

Gelation of the polymer membranes using liquid electrolyte solutions (i.e., PS-free sol and PS-containing sol) was achieved to finally obtain the desired Li^+^-conducting composite electrolytes. The transition from solid to gel-like systems was very easily and quickly attained with the proposed electrospun fiber mats due to their high surface-to-volume ratio. Thermal properties of the resulting electrolytes have been checked after the gelation process. The DSC response of the new gelled electrolytes is reported in Figure 4. As expected, the melting transition of the polymer was highly influenced by the presence of the liquid component. The crystallinity of PEO was strongly reduced, almost suppressed, upon gelation, giving rise to an amorphous, plasticized electrolyte system. In this respect, the nature of the electrolyte solution, with or without polysulfide, appeared almost irrelevant. The main role was due to the ionic liquid (i.e., the major component of the liquid electrolytes), which interacted with the polymer chains, thus preventing their crystallization. Interestingly, a certain degree of crystalline phase, even though very small, was preserved when silica particles were present (see the melting peak around 60 °C in Figure 4b). This is quite reasonably attributed to preferential interactions established between the inorganic filler and the liquid solution, leaving partially-coordinated PEO chains free to crystallize. In this respect, if we assume the starting PEO powder as a reference, it was possible to compare the crystallinity of our different samples by dividing the enthalpy change for the melting transition of each quoted sample by the enthalpy of melting related to the PEO powder. As already pointed out, crystallinity of the electrospun starting membranes was higher than that of the PEO powder due to the ordering effect of the electrospinning process [3], i.e., 1.81 times higher for the es-PEO membrane and 1.44 times higher for the es-PEO-SiO_2_ membrane. Such an estimate was not possible for silica-free gelled systems, as no melting was observed in the DSC traces. Whereas, a very low crystalline degree was maintained in the silica-added gel polymer electrolytes, with the crystallinity being 0.12 and 0.08 times that of PEO powder for es-PEO-SiO_2_/PS-free sol and es-PEO-SiO_2_/PS-containing sol, respectively. Another thermal response was noticed around −80 °C in the DSC traces of Figure 4 due to the glass transition of the ionic liquid component [22].

**Figure 5:**
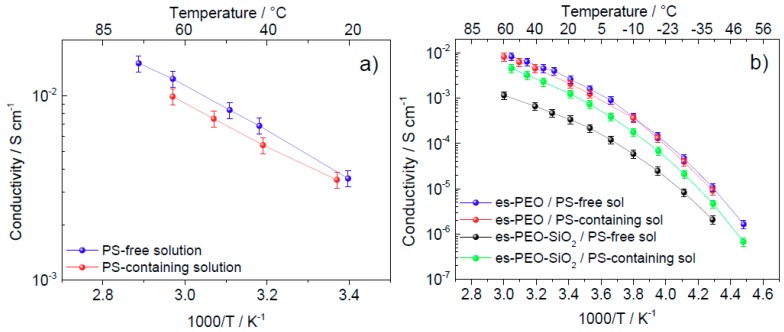
Arrhenius plots of conductivity of the swelling liquid solution (**a**) and of the gelled electrolyte membranes (**b**).

Functionality of the gelled membranes as electrolyte was tested using EIS measurements performed at increasing temperature in the range −50 °C to 60 °C. For comparison purposes, impedance spectra were recorded for the liquid electrolyte solutions as well, in this case limiting the temperature range between 20 °C and 70 °C. Conductivity values, obtained from the impedance spectra in the investigated T-ranges, are reported in Figure 5a,b for the swelling solutions and for the swollen membranes, respectively, in the form of Arrhenius plots. Conductivity values, extrapolated from the plots in Figure 5b at two temperatures of interest (i.e., 25 °C and 50 °C, representing normal operating conditions and melting region of the polymer, respectively) are reported in Table 2.

With the exception of the es-PEO-SiO_2_/PS-free sol sample, very high conductivities were achieved for all the gelled systems at both investigated temperatures. It is worth noticing that these room-temperature σ values are typical of viscous organic electrolytes used in lithium-batteries, revealing that the polymer membranes here were very well plasticized. As shown below, a very limited conductivity decrease was observed when moving from the liquid electrolytes, PS-free sol and PS-containing sol, to the gelled polymer systems. As expected, a temperature-activated transport mechanism was found, giving higher conductivity values at 50 °C with respect to 25 °C. 

In Figure 5a, it is possible to observe both the liquid electrolytes showing interesting ionic conductivity, and the presence of polysulfide ions (in PS-containing solution) seemed to affect the conduction properties very little. Overall, the detected conductivity values appear suitable for battery applications in a wide temperature range. The addition of polysulfide in solution has, in general, two opposite effects on the overall ionic conductivity: on one hand, the number of charged species increased due to the intake of anions in the solution; on the other hand, an increase in viscosity is expected, which hindered the ion mobility. In our systems, the two solutions had very high, similar conductivity values, indicating that these effects offset each other.

As shown in Figure 5b, conductivity was lower for the silica-added gel polymer electrolytes (black and green plots) compared to the silica-free systems (blue and red plots). This can be explained by considering a possible retention of the liquid electrolyte fraction on the silica particles, hindering the polymer chain-assisted ion transport. In this respect, a certain role is played by the nature of the liquid electrolyte. Indeed, big differences were observed among the two silica-added membranes according to the type of swelling liquid electrolyte, with the polysulfide-doped solution giving higher overall conductivity with respect to the polysulfide-free system (compare es-PEO-SiO_2_/PS-containing sol with es-PEO-SiO_2_/PS-free sol in Figure 5b).

Each curve of Figure 5b was fitted using the Vogel–Tamman–Fulcher (VTF) equation:

$$\sigma \left(T\right)=A\cdot T^{-\frac{1}{2}}\cdot e^{-\frac{E_{A}}{R\left(T-T_{0}\right)}}$$

with *A* and *E_A_* parameters representing the charge carrier number and the activation energy, respectively, whereas *T*_0_ is the ideal glass transition temperature. Very high correlation (higher than 0.99) was found between the experimental and fitted curves, revealing that our systems followed the typical behavior of ion-conducting amorphous matrices where the polymer component assisted the ion transport. The extrapolated parameters were considered and reported in Table 3.

E_A_ values were low, compared to other IL-added PEO-based ion-conducting systems [23], meaning that ion transport and conduction mechanism were very easily activated. Moreover, no substantial differences were found among samples in terms of activation energy. Similarly, comparably low values of T_0_ were obtained, proving that all the gelled electrolytes exhibited amorphous behavior. Differences of relevance were found in terms of the A parameter. The smallest value was obtained for es-PEO-SiO_2_/PS-free sol sample, revealing that its low conductivity (see black curve in Figure 5b and values in Table 2) was actually due to a reduced number of total charge carriers. This supports our hypothesis that silica particles absorbed the liquid electrolyte, thus limiting the concentration of ions available for transport. Based on the higher conductivity and A values observed in es-PEO-SiO_2_/PS-containing sol, we conclude that the polysulfide opposes this retention effect of SiO_2_ additive to the advantage of the transport mechanism.

## 4. Conclusions

An easy way to obtain highly conductive gel polymer electrolytes was proposed in this paper. The swelling ability of high surface area, electrospun PEO membranes was exploited for the absorption of stable IL-based electrolytes. The gelling procedure, in terms of solid-to-liquid weight ratio (i.e., PEO membrane: IL-based electrolyte), was optimized to obtain a reproducible and high swelling degree. The absorption of the liquid component into the PEO membrane strongly reduced or even suppressed the crystallinity of the polymer, giving rise to amorphous, well-plasticized electrolyte systems. The effect of a silica particle additive, dispersed in the polymer matrices during the electrospinning process, was also investigated. A certain degree of crystalline phase, even though very small, was preserved in the membranes after gelling when silica was present, which was attributed to preferential interactions between the inorganic filler and the liquid solution, leaving partially-coordinated PEO chains free to crystallize. This was reflected in the conducting properties of the gel polymer electrolytes, showing lower ion conductivity in the silica-added sample because of a reduced number of available charge carriers.

In view of possible applications in lithium-sulfur batteries, the addition of a polysulfide component (i.e., Li_2_S_8_) in the swelling solution was considered. Such a polysulfide affected the ionic conductivity of the silica-free gel polymer electrolytes very little. On the contrary, it had a beneficial effect in the presence of silica. Apparently, the polysulfide opposed the liquid retention of SiO_2_ particles to the advantage of the transport mechanism.

Overall, selected compositions proposed here showed conductivity values suitable for battery applications in a wide range of temperatures. All these findings address the potentiality of such gelled electrolytes. 

## Figures and Tables

**Table 1:** Enthalpy change and temperature related to PEO melting (values derived from DSC response in Figure 3).

Sample	∆H_melting_/J·g^−1^	T_melting_/°C
**PEO powder**	104.0	72.3
**es-PEO membrane**	188.5	68.7
**es-PEO-SiO_2_ membrane**	149.7	72.2

**Table 2:** Conductivity values extrapolated at 25 °C and 50 °C for the gelled electrolyte membranes.

Sample	σ_T = 25 °C_ (S/cm)	σ_T = 50 °C_ (S/cm)
**es-PEO/PS-free sol**	3.3 × 10^−3^	7.2 × 10^−3^
**es-PEO/PS-containing sol**	2.5 × 10^−3^	6.1 × 10^−3^
**es-PEO-SiO_2_/PS-free sol**	4.0 × 10^−4^	8.8 × 10^−4^
**es-PEO-SiO_2_/PS-containing sol**	1.5 × 10^−3^	3.7 × 10^−3^

**Table 3:** Parameters of the VTF equation derived by fitting the curves in Figure 5b.

Sample	E_A_ (kJ/mol)	T_0_ (K)	A (S/cm)
es-PEO/PS-free sol	6.76 ± 0.4	164.3 ± 2.5	23.27 ± 5.67
es-PEO/PS-containing sol	5.92 ± 0.3	170.5 ± 2.3	11.84 ± 2.38
es-PEO-SiO_2_/PS-free sol	5.05 ± 0.2	170.7 ± 1.9	1.24 ± 0.18
es-PEO-SiO_2_/PS-containing sol	7.00 ± 0.1	163.6 ± 0.8	13.80 ± 1.06
